# Predictive Value of C-reactive Protein in the Spontaneous Passage of Lower Ureteric Stones: A Prospective Single-Centre Study

**DOI:** 10.7759/cureus.62669

**Published:** 2024-06-19

**Authors:** Ahsan Ahmad, Khalid Mahmood, Nandesh Kumar, Ravi R Sharma, Nikhil Ranjan, Rakesh Kumar Anand, Shishir Kumar

**Affiliations:** 1 Urology, Indira Gandhi Institute of Medical Sciences, Patna, IND; 2 Community Medicine, Indira Gandhi Institute of Medical Sciences, Patna, IND

**Keywords:** c –reactive protein (crp), ureteric stone, neutrophil levels, leukocyturia, biomarker

## Abstract

Introduction: Ureteric stones, characterised by their presence in the ureter, present a common yet often painful urological condition requiring timely intervention. As C-reactive protein (CRP) emerges as a potential biomarker, its correlation with the spontaneous stone passage (SSP) offers valuable insights into patient management and treatment strategies. The present study aimed to assess if CRP levels can predict SSP in symptomatic lower ureteric calculi of size 5 mm-10 mm.

Materials and methods: This prospective observational study, conducted at the Indira Gandhi Institute of Medical Sciences in Patna, India, from July 2022 to June 2023, focused on individuals aged 13 to 60 years presenting with ureteric colic and single distal ureteral stones (5 mm-10 mm). Patients underwent comprehensive initial assessment and monitoring, including diagnostic procedures such as a complete blood count, urinalysis, CRP levels, and renal function evaluations. Treatment consisted of hydration encouragement, tamsulosin (0.4 mg) daily administration, and diclofenac (50 mg) as needed. Follow-up assessments at one-month post-treatment involved clinical examination and imaging studies to evaluate treatment efficacy.

Results: This study analysed 157 patients with ureteric stones, finding that 76% experienced SSP. Lower CRP levels (≤6 mg/L), along with other laboratory parameters like low white blood cell counts, low neutrophil levels, absence of leukocyturia, absence of hematuria, and lower urine specific gravity, were associated with higher SSP rates. C-reactive protein levels ≤6 mg/L emerged as a strong predictor of SSP in multiple regression analysis.

Conclusion: The findings underscore the potential utility of CRP as a predictive biomarker in guiding the management and treatment strategies for ureteric stones.

## Introduction

Urinary tract stones are the third most common urinary tract disorder, following urinary tract infections and prostatic pathologies such as benign prostatic hyperplasia and prostate cancer [1-3]. Urinary stones are common worldwide, with a prevalence of about 12% [4]. Their prevalence in India also reflects worldwide prevalence, which stands at approximately 12% and is relatively more common in the northern part of India, where it is 15% [5]. Due to their substantial impact on the severity of the disease and the deterioration of renal function [6], it is crucial to ensure attentive management for individuals dealing with urinary stones. 

Treatment decisions are typically based on stone size, location, composition, patient symptoms, and overall clinical status [7]. Various treatment options are available for ureteric stones, such as watchful waiting, medical expulsive therapy, ureteroscopy and stone removal, extracorporeal shock wave lithotripsy (ESWL), and open surgery. Conservative or expected management can sometimes result [8].

Although conservative management is simple and cost-effective, it carries potential risks such as non-passage of stones, leading to bothersome symptoms such as pain, fever, urinary tract infection, renal function deterioration, and sepsis [6, 9-12]. In addition, guidelines advocate conservative approaches for uncomplicated ureteral stones <10 mm; however, watchful waiting may lead to urosepsis and renal impairment. Therefore, identifying predictors for spontaneous passage or complications is crucial [13]. 

In the quest for ideal biomarkers readily accessible to urological centres managing renal colic patients, attention has turned to routinely collected and affordable blood tests. White blood cell (WBC), neutrophil count, and C-reactive protein (CRP) are blood tests that are routinely performed in patients on admission with acute ureteric colic and have attracted attention for their potential to assess the severity of inflammation [14]. These biomarkers aid clinicians in diagnosing and managing acute ureteric colic, guiding treatment decisions, and monitoring the patient's response to therapy [15]. Stones cause inflammation of the ureteric wall [16]. This inflammation causes a rise in different markers such as CRP, total leukocyte count, neutrophil count, neutrophil-to-lymphocyte ratio (NLR), and procalcitonin values [17].

C-reactive protein was discovered by Tillett and Francis in 1930 [18]. It is an acute-phase reactant protein that is primarily induced by the interleukin-6 (IL-6) action of the gene responsible for the transcription of CRP during the acute phase of an inflammatory/infectious process. It can be elevated in ureteric colic secondary to ureteral mucosal inflammation [19]. It shows a distinct association with the pathological conditions of kidney disease, functioning as a biomarker for renal pathology. It has been explored in a urological context, including assessing renal injury in pyelonephritis and urinary tract infection severity in children. Additionally, it may help avoid unnecessary procedures like micturating cystourethrograms in paediatric patients with vesicoureteric reflux and fever [20,21].

Therefore, a comprehensive study was conducted, examining factors like WBC count, neutrophil count, urine specific gravity, leukocyturia, hematuria, and indicators, primarily CRP, to determine their predictive value in the spontaneous passage of lower ureteric stones. The study specifically aimed to assess CRP levels that could predict spontaneous stone passage (SSP) in symptomatic lower ureteric calculi.

## Materials and methods

Study design

This prospective observational study took place at the urology department of the Indira Gandhi Institute of Medical Sciences in Patna, India, over one year, from July 2022 to June 2023, involving patients from both outpatient and inpatient wards. The study was conducted in accordance with the Declaration of Helsinki and approved by the Institutional Ethics Committee of the Indira Gandhi Institute of Medical Sciences, Patna (approval No. 527/IEC/IGIMS/2022). Written informed consent was obtained from all the participants prior to enrollment in this study.

Inclusion and exclusion criteria

The study included patients aged between 13 to 60 years with ureteric colic who had a single distal ureteral stone ranging from 5 mm to 10 mm in diameter. Exclusion criteria comprised patients with severe hydronephrosis, symptomatic urinary system infection, acute renal failure, congenital ureteral anomaly, history of ureteral stenosis or reconstructive ureteral surgery, ureteroscopy and stone removal, chronic inflammatory disease (like rheumatoid arthritis, ankylosing spondylitis, or ulcerative colitis), active neoplastic disease, active infective disease, and thyroid disease. Patients who were on anti-inflammatory or anti-microbial drugs or immunosuppressive treatment were excluded from this study. Pregnant women and patients not compliant with medical expulsive therapy (MET) or needing some form of intervention were also excluded from the study. 

Collection of medical history and follow-up

Patients experiencing ureteric colic underwent initial evaluation and subsequent monitoring. Laboratory tests conducted during the initial visit encompassed a complete blood count, urinalysis, serum CRP levels, and renal function assessments. Imaging procedures such as X-ray of the kidney, ureter, and bladder (KUB), ultrasonography (USG) of KUB, and intravenous urography or computed tomography urography (CTU) were done. Patients enrolled in the study were advised to consume 2.5 litres of water daily and were prescribed tamsulosin (0.4 mg) once daily at bedtime and diclofenac (50 mg) as needed. Stone dimensions were recorded. Factors such as age, sex, smoking history, body mass index (BMI), stone size and location, serum WBC, and neutrophil count were documented. Stone composition analysis was performed whenever available. Follow-up assessments were conducted one month post-treatment, including clinical examination and imaging studies like X-ray KUB or non-contrast CT KUB.

Study endpoints

The primary objective of the study was to evaluate CRP as a predictor for SSP in symptomatic lower ureteric calculi while also investigating the correlation between CRP levels and the success of MET. Additionally, the secondary objective aimed to assess the impact of various factors, such as stone size, CRP levels, leukocytosis, leukocyturia, urinary specific gravity, hematuria, and other variables, on the probability of experiencing SSP.

Statistical analysis

Statistical analysis was assessed using IBM SPSS Statistics for Windows, version 23.0 (IBM Corp., Armonk, NY). Descriptive statistics were used to describe categorical variables (frequency and percentages) and continuous variables (mean and standard deviation (SD) or median and range (depending on the normality of data)). The comparison of qualitative variables between the groups was done using an independent sample T test and a Mann-Whitney U test for parametric and non-parametric variables, respectively. A quantitative comparison between the groups was done using the chi-square test. Multiple binary logistic regressions were performed to identify covariates associated with SSP. A p-value of <0.05 was considered statistically significant. 

## Results

Demographic data

A total of 188 patients who met the inclusion criteria were enrolled in the study; however, 31 of them discontinued participation for various reasons. Then the remaining 157 patients were studied. Out of the 157 patients in the study, 120 patients (76%) had an SSP, while 37 patients (24%) had non-spontaneous passage (NSP). The mean age of patients in the NSP group was higher compared to patients in the SSP group (33.0 vs. 31.5 years, respectively). The majority of patients were men in both groups (66.0% and 76.0%). The average BMI in the SSP group was 24.65 kg/m², while in the NSP group, it was 26.47 kg/m², with no statistically significant difference (p = 0.598). No significant difference was observed in terms of side with the stone and smoking history between both groups (p = 0.75 and p = 0.086, respectively). The majority of SSPs were of the calcium oxalate type (58%), followed by infective stone (10%), calcium phosphate (10%), and uric acid (2%). In 20% of cases, no stone analysis could be done (Table 1).

**Table 1:** A comparison of the patient characteristics of the SSP and NSP groups Data are presented as n (%) unless otherwise specified. BMI: body mass index; CRP: c-reactive protein; SSP: spontaneous stone passage; NSP: non-spontaneous passage; ; SD: standard deviation; RBC: red blood cell;  WBC: white blood cell; HPF: high power field

Characteristics	SSP group (n=120)	NSP group (n=37)	P-value
Age (years), mean (SD)	31.5 (6.8)	33.0 (9.0)	0.014
Sex			0.261
Men	79 (66.0)	28 (76.0)	
Women	41 (34.0)	9 (24.0)	
BMI (kg/m^2^), mean (SD)	24.65 (2.4)	26.47 (1.8)	0.598
Smoking history	31 (26.0)	15 (40.0)	0.086
Side with the stone			0.75
Right	64 (53.0)	21 (57.0)	
Left	56 (47.0)	16 (43.0)	
Stone analysis			-
Calcium oxalate	70 (58.0)	-	
Infection stone	12 (10.0)	-	
Calcium phosphate	12 (10.0)	-	
Uric acid	2 (2.0)	-	
No stone analysis	24 (20.0)	-	
CRP (mg/L)			<0.001
≤6	91 (76.0)	4 (11.0)	
>6	29 (24.0)	33 (89.0)	
WBC (10^3^/µL)			0.016
<10	98 (82.0)	22 (59.0)	
10-15	18 (15.0)	11 (30.0)	
>15	4 (3.0)	4 (11.0)	
Neutrophil (%)			<0.001
50-75	110 (92.0)	22 (60.0)	
˃75	10 (8.0)	15 (40.0)	
Serum creatinine (mg/dL), median (range)	0.8 (0.6-1.1)	1.1 (0.8-1.4)	<0.001
Leukocyturia	22 (18.0)	17 (46.0)	0.001
Hematuria			<0.001
Microscopic	18 (15.0)	22 (60.0)	
Gross	1 (1.0)	5 (13.0)	
Urine specific gravity			<0.001
≤1.008	96 (80.0)	15 (40.0)	
>1.008	24 (20.0)	22 (60.0)	

Laboratory parameters 

Among patients with a CRP level of ≤6 mg/L, 91 (76.0%) patients experienced SSP, while four patients (11.0%) did not. The proportion of patients with CRP levels >6 mg/L was significantly higher in the NSP group compared to the SSP group (89.0% vs. 24.0%; p <0.001). Around 41.0% of patients from the NSP group and 18.0% from the SSP group had a WBC of >10,000 cells/mm^3^ (p = 0.016). The majority of patients with NSP had leukocyturia compared to those in the SSP group (46.0% vs. 18%; p <0.001). The NSP group had a higher proportion of patients with a neutrophil count >75% compared to the SSP group (40% vs. 8.0%; p <0.001). The median serum creatinine in the SSP group was 0.8, whereas in the NSP group, it was 1.1 (0.8-1.4), indicating a statistically significant distinction (p <0.001). Leukocyturia was noted in a lower percentage among the SSP group (18.0%) compared to a significantly higher proportion within the NSP group (46.0%), showing a significant difference (p = 0.001). Microscopic and gross hematuria were significantly more prevalent in the NSP group in comparison to the SSP group (60.0% and 13.0% vs. 15% and 1.0%, respectively). A total of 80.0% of patients from SSP and 40.0% of participants from NSP had a specific gravity ≤1.008, while 60.0% of participants from the NSP group and only 20.0% of participants from the SSP group had a specific gravity >1.008. This difference was significant (p <0.001) (Table 1).

Comparison between the stone size of patients among the SSP and NSP groups

The patients in the NSP group had a higher median stone size than those in the SSP group (8.2 mm vs. 5.4 mm). The majority of patients (72.0%) in the SSP group had stone sizes ranging from 5 mm-5.9 mm, while 43.0% of patients in the NSP group had stone sizes ranging from 8 mm-8.9 mm, with a significant difference (p <0.001) (Table 2).

**Table 2:** Comparison of stone size between the SSP group and the NSP group Data are presented as n (%). SSP: spontaneous stone passage; NSP: non-spontaneous stone passage

Variables	SSP group (n=120)	NSP group (n=37)	P-value
Stone size, median	5.4	8.2	-
Stone size group (mm)			
5-5.9	86 (72.0)	2 (5.0)	<0.001
6-6.9	16 (13.0)	3 (8.0)	
7-7.9	11 (9.0)	4 (11.0)	
8-8.9	5 (4.0)	16 (43.0)	
9-9.9	2 (2.0)	7 (19.0)	
10	-	5 (14.0)	

Multiple regression analysis 

Patients with CRP ≤6 mg/L showed approximately 26 times higher chances of developing SSP (p <0.001). Similarly, those with a WBC <10,000 cells/mm^3^ were four times more likely to experience SSP (p = 0.045) compared to those with counts >15,000 cells/mm^3^ (p = 0.540). Neutrophil levels ≤75% were significantly associated (p <0.001) and showed approximately eight times higher SSP. Low serum creatinine levels were associated considerably with SSP (P<0.001). If no leukocyturia was found, then SSP was significant (p <0.001). Hematuria was associated with a significantly lower SSP (P<0.001). Urine specific gravity ≤1.008 showed approximately six times higher SSP rate (p <0.001). As the stone size increased, the rate of SSP decreased (p <0.001). The lower age group had more chances of SSP and an increase in age was associated with lower SSP significantly (p <0.05). Patients with a lower BMI (18.5-24.9 kg/m^2^) were significantly associated with SSP, approximately four times more than others. Conversely, factors such as sex, the side with the stone, and smoking history did not exhibit a significant association with SSP (Table 3).

**Table 3:** Patient characteristics associated with SSP by using multiple binary logistic regression model B: coefficient estimates from the regression equation; Df: degree of freedom; Exp: exponential; SE: standard error; Wald: a reference to the Wald test; CRP: C-reactive protein; WBC: white blood cell;  SSP: spontaneous stone passage

Parameters	B	SE	Wald	Df	Exp (B)	P-value
Age groups (years)						
21-30	2.996	1.612	3.452	1	20.000	0.063
31-40	2.100	1.266	2.750	1	8.167	0.097
41-50	2.024	1.261	2.577	1	7.571	0.108
>50	0.560	1.330	0.177	1	1.750	0.674
Sex 1	-0.479	0.429	1.248	1	0.619	0.264
CRP ≤6 mg/L	3.254	0.571	32.498	1	25.888	0.000
WBC <10000 cells/mm^3^	1.494	0.745	4.017	1	4.455	0.045
WBC >15000 cells/mm^3^	0.492	0.804	0.375	1	1.636	0.540
Neutrophil ≤75%	2.015	0.470	18.353	1	7.500	<0.001
Serum creatinine (mg/dL)	-12.061	2.074	33.826	1	0.000	<0.001
Side of stone	-0.120	0.379	0.101	1	0.887	0.751
BMI group (kg/m^2^)						
18.5-24.9	1.427	1.230	1.346	1	4.167	0.246
25-29.9	-0.762	1.179	0.418	1	0.467	0.518
Absence of leukocyturia	1.331	0.406	10.777	1	3.786	0.001
Absence of microscopic hematuria	3.922	1.145	11.743	1	50.500	0.001
Absence of gross hematuria	1.409	1.141	1.525	1	4.091	0.217
Specific gravity ≤1.008	1.769	0.405	19.064	1	5.867	<0.001
Stone analysis						
Calcium oxalate	-1.702	0.766	4.934	1	0.182	0.026
Infection stone	0.000	1.275	0.000	1	1.000	1.000
Calcium phosphate	-0.693	1.061	0.427	1	0.500	0.513
Uric acid	18.718	28420.722	0.000	1	1346229	0.999
Smoking history	0.672	0.394	2.899	1	1.957	0.089
Stone size (mm)						
6-6.9	-2.087	0.953	4.801	1	0.124	0.028
7-7.9	-2.750	0.923	8.868	1	0.064	0.003
8-8.9	-4.924	0.880	31.325	1	0.007	<0.001
9-9.9	-5.014	1.074	21.776	1	0.007	<0.001
10	-24.964	17974.843	0.000	1	0.000	0.999

## Discussion

Oedemas resulting from the impaction of a ureteric stone lead to swelling, infection, and spasms in the area distal to the stone, affecting the natural passage [22]. Conservative management can be very successful and cost-effective. Stones sized 5 mm or less have up to 98% spontaneous expulsion with conservative management, according to the American Urological Association [11]. Coll et al. studied and found a 60% passage rate for stone sizes of 5 mm-7 mm, 48% for 7 mm-9 mm, and 25% for more than 9 mm [23]. If stones remain in the same position for an extended period, they can cause mucosal oedema and fibrosis due to prolonged physical pressure or reduced blood flow to the affected area. Thus, a conservative approach can have morbid risks such as recurrent colic, infection, sepsis, and deranged renal functions. These approaches should have proper monitoring so that timed intervention can be done and serious complications can be avoided. Therefore, different inflammatory and biochemical markers are used, which can predict the fate of stones, and accordingly, treatment options can be given.

The decision between invasive techniques and conservative management poses a significant challenge in urology, prompting several studies to investigate potential predictive factors for the natural passage of ureteral stones in patients [24]. The present study examined the potential of CRP levels to predict SSP in symptomatic lower ureteric calculi sized between 5 mm and 10 mm. The key observations of the study are: a) lower CRP levels (≤6 mg/L), along with other factors like low WBC count, low neutrophil levels, absence of leukocyturia, absence of hematuria, and specific gravity ≤1.008, were associated with higher SSP rates; b) multiple regression analysis identified CRP levels ≤6 mg/L, lower WBC count, neutrophil levels ≤75%, absence of leukocyturia, absence of hematuria, and specific gravity ≤1.008 as independent predictors of SSP.

There was a significant contrast in mean age between the two groups, with the NSP group having a higher mean age of 33 years (p = 0.014). Age distribution revealed that the majority of patients were within the 31-40 age groups, with other age groups having varying representation. Similarly, Hamid et al. [25] categorised the patients based on age distribution, revealing that 45.7% belonged to the 18-30-year age group, another 45.7% were from the 31-45-year age group, and the remaining 18.7% were aged 46-60 years. In another study, 44.9% belonged to the 18-30-year age group, 46.4% to the 31-45-year age group, and the remaining 18.7% were aged 46-60 years [26]. Across these studies, the majority of patients fell within the 20-45 age range. In multiple studies, age and sex did not show notable variances [27-31]. Furthermore, Ozcan et al. [24] observed a higher rate of SSP in the younger age category, with no significant distinctions in sex between the groups, mirroring our findings. Sex did not exhibit any significant associations (p = 0.261). Thus, age may be a relevant factor to consider when predicting stone passage outcomes, with younger patients potentially exhibiting a higher rate of SSP.

Recently, the serum CRP level has been studied as a possible predictor for SSP in patients with ureteral stones [22]. In the present study, raised CRP levels were inversely proportional to the SSP rates (p <0.001). Similarly, in a study conducted by Park et al. [22], patients with low CRP levels had a 94.1% stone passage rate, while those with high CRP levels had a 50% rate. High CRP levels suggest a lower likelihood of SSP [22]. Similar results were found in studies conducted by Abushama et al. [27], Ramaswamy et al. [28], Mohammed et al. [29], Angulo et al. [30], Deldadeh-Moghaddam et al. [31], and Aldaqadossi et al. [32].

The present analysis revealed a notable distinction (p <0.001) between patients in the NSP and SSP groups concerning the distribution of individuals with CRP levels ≤6 mg/L. Remarkably, the majority (76.0%) of SSP patients had CRP levels within this lower range, signifying a potential association between lower CRP levels and the likelihood of SSP. In contrast, only a small fraction (11.0%) of NSP patients exhibited CRP levels ≤6 mg/L, suggesting that lower CRP levels were predominantly observed in those who experienced SSP. Conversely, when considering CRP levels >6 mg/L, a distinct pattern emerged. The majority (89.0%) of NSP patients fell into this higher CRP category, indicating a likely association between elevated CRP levels and the absence of SSP. Conversely, only a minority (24.0%) of SSP patients had CRP levels >6 mg/L, indicating that higher CRP levels were more frequently observed in patients who did not experience SSP (p <0.001).

In the multiple binary logistic regression analysis, the likelihood of SSP was approximately 26 times higher in patients with CRP levels ≤6 mg/L compared to those with higher CRP levels (p <0.001). This significant association underscores the predictive value of CRP levels ≤6 mg/L in indicating SSP, suggesting its utility as a prognostic marker for favourable stone outcomes without the need for intervention.

Several other inflammatory and biochemical markers are used, which might predict the prognosis of stones, and accordingly, treatment options can be given. These markers include age, sex, WBC, neutrophil count, serum creatinine, side, size, and stone analysis, urine analysis, hematuria, and urine-specific gravity.

In the present study, a WBC count <10 (103/µL) was found to be significantly associated with a higher SSP (p = 0.016). Similarly, in multiple binary logistic analysis, a WBC <10,000 cells/mm^3^ was associated with a higher SSP (P<0.045), which also aligns with previous studies [27,33,34] proving that the lesser WBC count shows a higher passage of the stone. Similar results were found in studies conducted by Abushama et al. [27], Kumar et al. [33], and Timilsina et al. [34].

Neutrophils, a type of white blood cell with neutral-staining granules, increase in various conditions, like inflammatory response, bacterial infection, myocardial infarction, and burns. When there is a blockage due to a ureteral stone, neutrophil levels rise as part of the body's inflammatory response [22]. In a recent study, a neutrophil percentage of 75.0% or lower was found to significantly predict a nearly eightfold increase in the rate of SSP (p <0.001), also aligned with a few studies [22,33,34]. Similar results were found in studies conducted by Park et al. [22], Kumar et al. [33], and Timilsina et al. [34]. But contrary to the present study, Sfoungaristos et al. showed the increased spontaneous passage of stones in patients with a raised WBC count and neutrophil count [17].

Patients who have stones may have a higher likelihood of undergoing serum creatinine measurement to properly evaluate kidney function and assess for any potential renal impairment or damage caused by the stones or associated complications such as obstruction or infection [35]. The SSP group significantly had serum creatinine in the lower range compared to the NSP group (p <0.001). Similarly, multiple binary logistics regression analysis shows low serum creatinine levels associated significantly with SSP (p <0.001). However, Shah et al. [14] found no association between serum creatinine and SSP. 

Leukocyturia was found in a lower percentage of the SSP group (18%) and a significant percentage of the NSP group (46%; p = 0.001), and as per multivariate analysis, lower leukocyturia shows (p <0.001) approximately four times higher passage of the stone. Cilesiz et al. [36] also observed a significant association between leukocyturia and less SSP (p = 0.004). Therefore, the level of leukocyturia can serve as a predictor of the presence of NSP.

Microscopic hematuria is diagnosed when ≥3 red blood cells (RBCs) are observed per high-power field (HPF) during urine microscopic analysis, while gross hematuria involves visibly passing blood in urine and is confirmed through microscopic examination [37]. In the current study, microscopic and gross hematuria were found significantly more in the NSP group (60.0% and 13.0%) in comparison to the SSP group (15.0% and 1.0%). Through a literature search, 49 articles involving 15,860 patients were identified. The combined prevalence of microhematuria for suspected acute renal colic was found to be 77.0% (95% CI: 73.0-80.0%) [38]. Therefore, hematuria can predict the failure of SSP.

A higher urinary specific gravity is significantly associated with a lower rate of SSP. Specifically, 60% of the NSP group had a specific gravity >1.008, and only 20% of the SSP group had a specific gravity >1.008 (p <0.001). In multiple binary logistic regression analysis, a specific gravity of ≤1.008 was significantly linked to a six-fold increase in the SSP rate (p <0.001).

In the present study, increasing stone size correlates with decreased SSP. These results reinforce the established trend of SSP decreasing as stone size increases, consistent with prior research [14, 27-31].

Several studies have explored the link between urinary system stones and serum CRP levels, along with other inflammatory markers [28, 30, 39]. Serum CRP measurement emerges as a promising new biomarker for predicting ureteral stone presence, offering potentially more accurate diagnostic insights.

While prospective studies offer valuable insights into causal relationships and minimise certain biases associated with retrospective designs, they are still susceptible to limitations. These may include challenges in participant recruitment, loss of follow-up, and potential biases introduced during data collection and analysis. Additionally, the generalizability of findings from a single prospective study may be limited, necessitating validation through replication in larger, more diverse cohorts. Furthermore, despite efforts to control for confounding variables, residual confounders may still exist, impacting the accuracy of the observed associations.

## Conclusions

Lower CRP levels (≤6 mg/L), along with other factors like demographics and lab results, suggest a better chance of naturally passing symptomatic lower ureteric stones (5 mm-10 mm). These results highlight CRP as a potentially useful marker for predicting and guiding the treatment of ureteric stones. Moreover, this study showed low WBC count, low neutrophil levels, absence of leukocyturia, absence of hematuria, and specific gravity ≤1.008 were associated with higher SSP rates.

## References

[1] Papadoukakis S, Stolzenburg JU, Truss MC (2006). Treatment strategies of ureteral stones. EAU-EBU Update Series.

[2] Schade GR, Faerber GJ (2010). Urinary tract stones. Prim Care.

[3] López M, Hoppe B (2010). History, epidemiology and regional diversities of urolithiasis. Pediatric Nephrol.

[4] Nojaba L, Guzman N (2023). Nephrolithiasis (Kidney Stones). Pediatr Nephrol.

[5] Guha M, Banerjee H, Mitra P, Das M (2019). The demographic diversity of food intake and prevalence of kidney stone diseases in the Indian continent. Foods.

[6] Ordon M, Andonian S, Blew B, Schuler T, Chew B, Pace KT (2015). CUA guideline: management of ureteral calculi. Can Urol Assoc J.

[7] McClinton S, Cameron S, Starr K (2018). TISU: extracorporeal shockwave lithotripsy, as first treatment option, compared with direct progression to ureteroscopic treatment, for ureteric stones: study protocol for a randomised controlled trial. Trials.

[8] Shafi H, Moazzami B, Pourghasem M, Kasaeian A (2016). An overview of treatment options for urinary stones. Caspian J Intern Med.

[9] Phillips E, Kieley S, Johnson EB, Monga M (2009). Emergency room management of ureteral calculi: current practices. J Endourol.

[10] Türk C, Petřík A, Sarica K, Seitz C, Skolarikos A, Straub M, Knoll T (2016). EAU guidelines on interventional treatment for urolithiasis. Eur Urol.

[11] Segura JW, Preminger GM, Assimos DG, Dretler SP, Kahn RI, Lingeman JE, Macaluso JN Jr (1997). Ureteral Stones Clinical Guidelines Panel summary report on the management of ureteral calculi. The American Urological Association. J Urol.

[12] Bailey HH, Love RJM (2018). Bailey & Love's Short Practice of Surgery, 27th Edition.

[13] Assimos D, Krambeck A, Miller NL (2016). Surgical management of stones: American Urological Association/endourological Society guideline, part I. J Urol.

[14] Shah TT, Gao C, Peters M (2019). Factors associated with spontaneous stone passage in a contemporary cohort of patients presenting with acute ureteric colic: results from the Multi-centre cohort study evaluating the role of Inflammatory Markers In patients presenting with acute ureteric Colic (MIMIC) study. BJU Int.

[15] Sfoungaristos S, Kavouras A, Katafigiotis I, Perimenis P (2012). Role of white blood cell and neutrophil counts in predicting spontaneous stone passage in patients with renal colic. BJU Int.

[16] Morgentaler A, Bridge SS, Dretler SP (1990). Management of the impacted ureteral calculus. J Urol.

[17] Sfoungaristos S, Kavouras A, Perimenis P (2012). Predictors for spontaneous stone passage in patients with renal colic secondary to ureteral calculi. Int Urol Nephrol.

[18] Tillett WS, Francis T (1930). Serological reactions in pneumonia with a non-protein somatic fraction of pneumococcus. J Exp Med.

[19] Pepys MB, Baltz ML (1983). Acute phase proteins with special reference to C-reactive protein and related proteins (pentaxins) and serum amyloid A protein. Adv Immunol.

[20] Dai J, Tang K, Xiao W (2014). Prognostic significance of C-reactive protein in urological cancers: a systematic review and meta-analysis. Asian Pac J Cancer Prev.

[21] Shoag J, Eisner BH (2014). Relationship between C-reactive protein and kidney stone prevalence. J Urol.

[22] Park CH, Ha JY, Park CH, Kim CI, Kim KS, Kim BH (2013). Relationship between spontaneous passage rates of ureteral stones less than 8 mm and serum C-reactive protein levels and neutrophil percentages. Korean J Urol.

[23] Coll DM, Varanelli MJ, Smith RC (2002). Relationship of spontaneous passage of ureteral calculi to stone size and location as revealed by unenhanced helical CT. AJR Am J Roentgenol.

[24] Özcan C, Aydoğdu O, Senocak C, Damar E, Eraslan A, Oztuna D, Bozkurt OF (2015). Predictive factors for spontaneous stone passage and the potential role of serum C-reactive protein in patients with 4 to 10 mm distal ureteral stones: a prospective clinical study. J Urol.

[25] Hamid H, Ali I, Ullah R (2022). Spontaneous expulsion of lower ureteric stones in patients with elevated level of C-reactive protein. Pak J Med Sci.

[26] Muhammad I, Shad N, Khan Khan (2022). Frequency of spontaneous stone expulsion of small lower ureteric stones in patients with raised serum CRP (C-reactive protein). J Khyber Coll Dent.

[27] Abushamma F, Ktaifan M, Abdallah A (2021). Clinical and radiological predictors of early intervention in acute ureteral colic. Int J Gen Med.

[28] Ramasamy V, Aarthy P, Sharma V, Thakur AP (2022). Role of inflammatory markers and their trends in predicting the outcome of medical expulsive therapy for distal ureteric calculus. Urol Ann.

[29] Mohammad E, Abbas KM, Hassan AF, Abdulrazaq AA (2018). Serum c-reactive protein as a predictive factor for spontaneous stone passage in patients with 4 to 8 mm distal ureteral stones. Int Surg J.

[30] Angulo JC, Gaspar MJ, Rodríguez N, García-Tello A, Torres G, Núñez C (2010). The value of C-reactive protein determination in patients with renal colic to decide urgent urinary diversion. Urology.

[31] Deldadeh-Moghaddam H, Nasrollahi S (2023). Relationship between spontaneous excretion of lower ureter stones with stone size and serum level of C-reactive protein. Int J Community Med Public Health.

[32] Aldaqadossi HA (2013). Stone expulsion rate of small distal ureteric calculi could be predicted with plasma C-reactive protein. Urolithiasis.

[33] Kumar A, Agrawal CS, Sah SP, Joshi BR, Gupta M (2017). Stone expulsion rate of small distal ureteric calculus could be predicted with serum C-reactive protein, white cell counts and neutrophil percentage: a prospective study. IOSR J Dent Med Sci.

[34] Timilsina BD, Karki O, Subedi Subedi, B B (2020). Correlation between expulsion of distal ureteric calculus with CRP level, WBC count and neutrophil percentage among the patients attending on Manipal Teaching Hospital, Pokhara (retrospective study). Nepal J Med Sci.

[35] Alexander RT, Hemmelgarn BR, Wiebe N (2012). Kidney stones and kidney function loss: a cohort study. BMJ.

[36] Nusret CC, Burak A, Can B, Öykü A, Bariş N (2021). The role of procalcitonin and other markers of inflammation in predicting spontaneous passage of ureteral stones. J Acad Res Med.

[37] Nagendra V, Dhande R, Mishra G, Reddy NG, Gowda H (2023). Hematuria as a sign of kidney stone disease evaluated using computed tomography: a review. Cureus.

[38] Minotti B, Treglia G, Pascale M, Ceruti S, Cantini L, Anselmi L, Saporito A (2020). Prevalence of microhematuria in renal colic and urolithiasis: a systematic review and meta-analysis. BMC Urol.

[39] Liang D, Liu C, Yang M (2024). The association between C-reactive protein levels and the risk of kidney stones: a population-based study. BMC Nephrol.

